# An *in vivo* biosensing, biomimetic electrochemical transistor with applications in plant science and precision farming

**DOI:** 10.1038/s41598-017-16217-4

**Published:** 2017-11-23

**Authors:** Nicola Coppedè, Michela Janni, Manuele Bettelli, Calogero Leandro Maida, Francesco Gentile, Marco Villani, Roberta Ruotolo, Salvatore Iannotta, Nelson Marmiroli, Marta Marmiroli, Andrea Zappettini

**Affiliations:** 1 Institute of Materials for Electronics and Magnetism (IMEM), National Research Council (CNR), Parco Area delle Scienze 37/A, 43124 Parma, Italy; 2 Department of Chemistry, Life Sciences and Environmental Sustainability, University of Parma, Parco Area delle Scienze, 11/A, 43100 Parma, Italy; 3 Department of Electrical Engineering and Information Technology, University Federico II, Naples, Italy; 4 Institute of Bioscience and Bioresources (IBBR), National Research Council (CNR), Via Amendola 165/A, 70126 Bari, Italy

## Abstract

The *in vivo* monitoring of key plant physiology parameters will be a key enabler of precision farming. Here, a biomimetic textile-based biosensor, which can be inserted directly into plant tissue is presented: the device is able to monitor, *in vivo* and in real time, variations in the solute content of the plant sap. The biosensor has no detectable effect on the plant’s morphology even after six weeks of continuous operation. The continuous monitoring of the sap electrolyte concentration in a growing tomato plant revealed a circadian pattern of variation. The biosensor has the potential to detect the signs of abiotic stress, and therefore might be exploited as a powerful tool to study plant physiology and to increase tomato growth sustainability. Also, it can continuously communicate the plant health status, thus potentially driving the whole farm management in the frame of smart agriculture.

## Introduction

The *in vivo*, *in loco* and real-time detection of qualitative and quantitative changes to a plant’s physiological state is of great relevance for the success of precision farming, crop management and plant phenotyping. This is especially relevant for tomato, a valuable horticultural crop, which represents an important dietary source of vitamins, minerals and fiber^1^. Commercially grown tomato requires the optimization of conditions throughout its cropping cycle, and thus would benefit from automated monitoring systems. However, few of the currently available platforms designed for the study of plant physiology are compatible with *in vivo* monitoring.

Several tools have been developed to study plant phenotype at subcellular, cellular, tissues, and organ level, up to the whole organism in all its dimensions^2^. Relative inexpensive and ubiquitous digital cameras have become widely used in high throughput phenotyping systems that utilized plant imaging to capture data^3^. The data acquisition is based on the sensor response at different light spectral ranges (400–1000 nm VIS, 700 nm NIR), nevertheless the detection is indirect and the sensors are sensible to relative broad ranges of electromagnetic spectrum so that many information can be lost in the output image^3^.

To understand how physiology and development are linked, and to visualize signaling processes in different organisms, two distinct classes of genetically encoded sensors have been developed and implemented, the first relies on the coupling of a ligand-sensitive receptor to a fluorescent protein, which is assayed by confocal microscopy; the second is based on electrochemical sensors combined with an enzymatic complex, which can be used to analyze liquid plant extracts^4–6^; among these, there are impedance sensors which measure variation in electrical resistance or capacitance. Receptors coupled to green fluorescent protein have been applied to monitor cellular concentrations of phytohormones^6–9^, of reactive oxygen species^10^, pH^11^, the calcium status^12^ and the presence of specific pathogens^13^. Sensors of this type have also been applied to explore the role of specific molecules in a given physiological process^14–19^, and have been used to reveal aspects of root cell metabolism^20,21^. Their use is, however, limited by a requirement for rather sophisticated equipment to detect and monitor the fluorescent signals. Electrochemical biosensors may also take advantage of a specific enzyme-substrate interaction. Their biggest advantage lies in the greater convenience of capturing electrical over optical signals. Applications include the monitoring of phytohormones accumulation^22,23^ and aspects of crop end-use quality^5^. Impedance-based sensors typically require electrodes of various shapes and composition^24–27^, which can lead to a localized disruption of cellular physiology^28^. A major drawback of this class of devices is that the chemical identity of the molecules producing the detected changes in the electrical output of the target remains unknown^24^.

Recently, plant tissues were successfully transformed in an electrochemical transistor opening new perspectives for bio-electronic applications^29^. The approach is based on the uptake of a conductive polymer through a cut plant stem which enables the transistor response but simultaneously induces a deep alteration of the plant structure, that drives ultimately to plant death. This paper has demonstrated that PEDOT:PSS based organic electrochemical transistors (OECT) have a high biocompatibility and may deeply interact with plant cellular system, even creating a transistor device inside their stem.

OECT-based sensors have shown high sensitivity^30,31^. When integrated onto a textile matrix, they have proved to be effective for monitoring the salt concentration of human sweat^32^, and have been designed to track adrenaline^33^ and tyrosine^34^. A great advantage of textile-based OECTs is their ability to absorb fluids, which allows for a simple means of monitoring cellular solutes. Moreover, textile functionalized threads are flexible and present a stable durable structure in aqueous environment. Here, the introduction of a biocompatible textile OECT into a tomato plant, termed a “bioristor”, is described. The resulting biosensor is readily integrated into the plant tissue, and is stable in the growing environment.

## Results

### Real time monitoring using the bioristor

**Figure 1:**
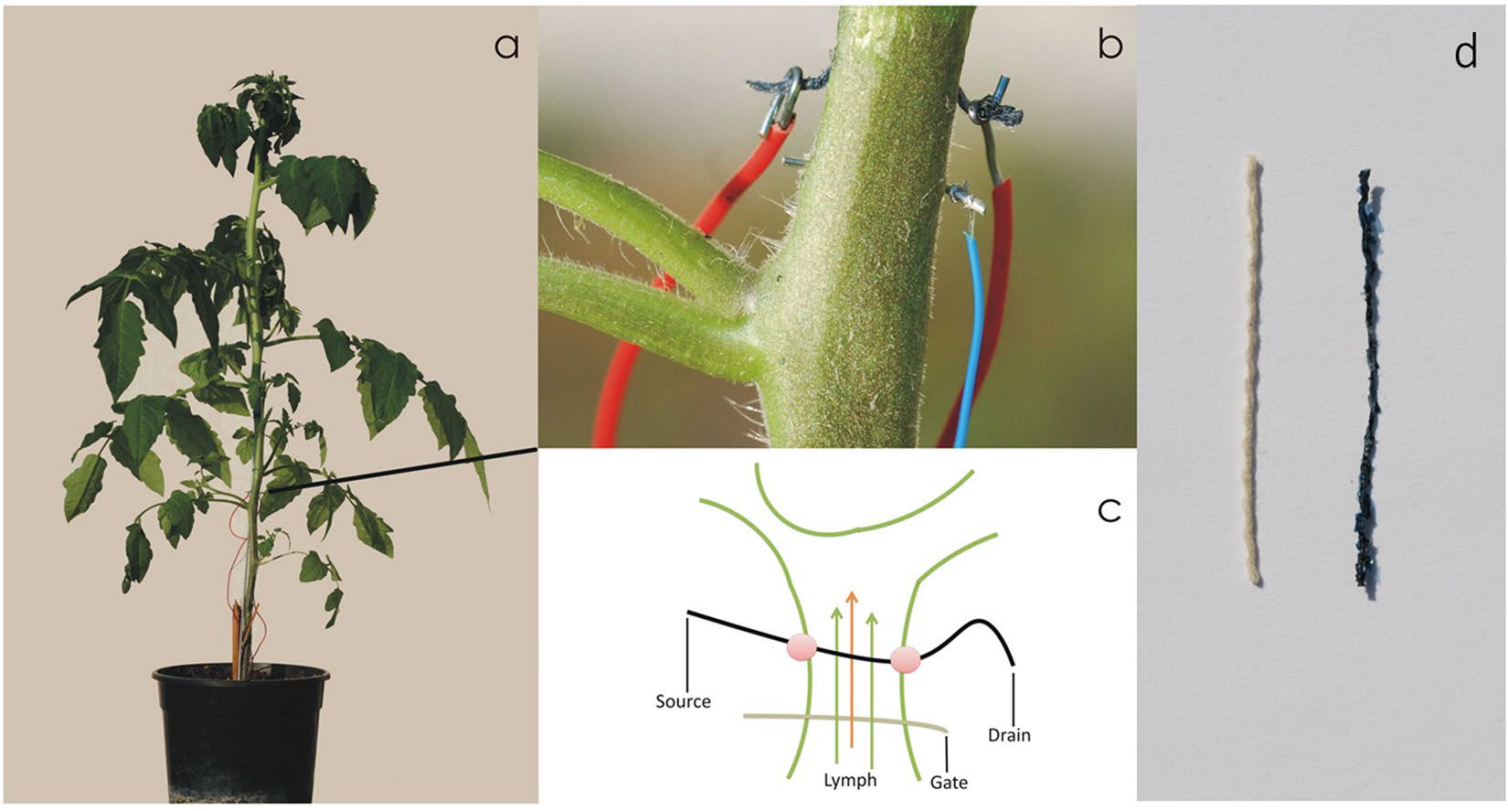
(**a**) A bioristor integrated in a tomato plant. (**b**) Detail of the textile device implantation and the silver gate connected through the plant stem. (**c**) Sketch of the proposed biosensor device showing the electrical connections Green lines: sketch of plant stems. Black line: textile thread. Grey line: gate electrode. Arrows: lymph flow. (**d**) Cotton thread untreated (left) and functionalized with Pedot:PSS (right).

The bioristor consists of a natural textile fiber functionalized by a conductive polymer^32^, directly integrated into the plant stem, and a thin silver wire to act as a gate electrode (Fig. 1). The thread presents a non-planar biomimetic surface, with a natural roughness at different micro and nano scale level.

**Figure 2:**
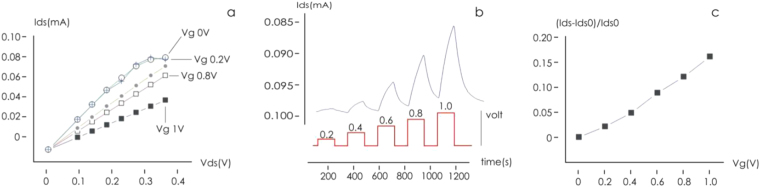
(**a**) The sensor characteristic of the device mounted on tomato plant 1. The proposed device behaves as an organic electrochemical transistor (OECT). (**b**) Sensor I_ds_ current at fixed V_ds_ for different V_gs_ bias (red). (**c**) Sensor response calculated from (**b**) for the different gate bias.

**Figure 3:**
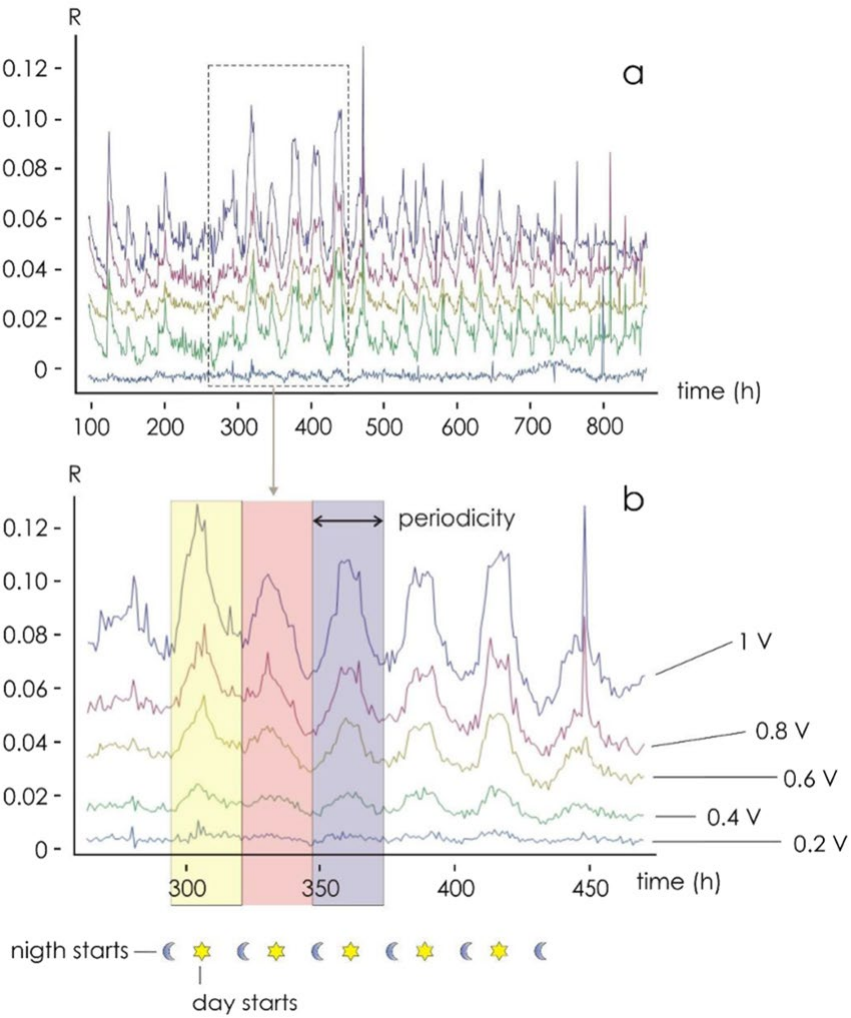
Sensor response (R) over 22 days (**a**) response at different V_gs_ bias; (**b**) detail of the sensor response for different gate voltages. Colored overlay highlights three day/night cycles.

**Figure 4:**
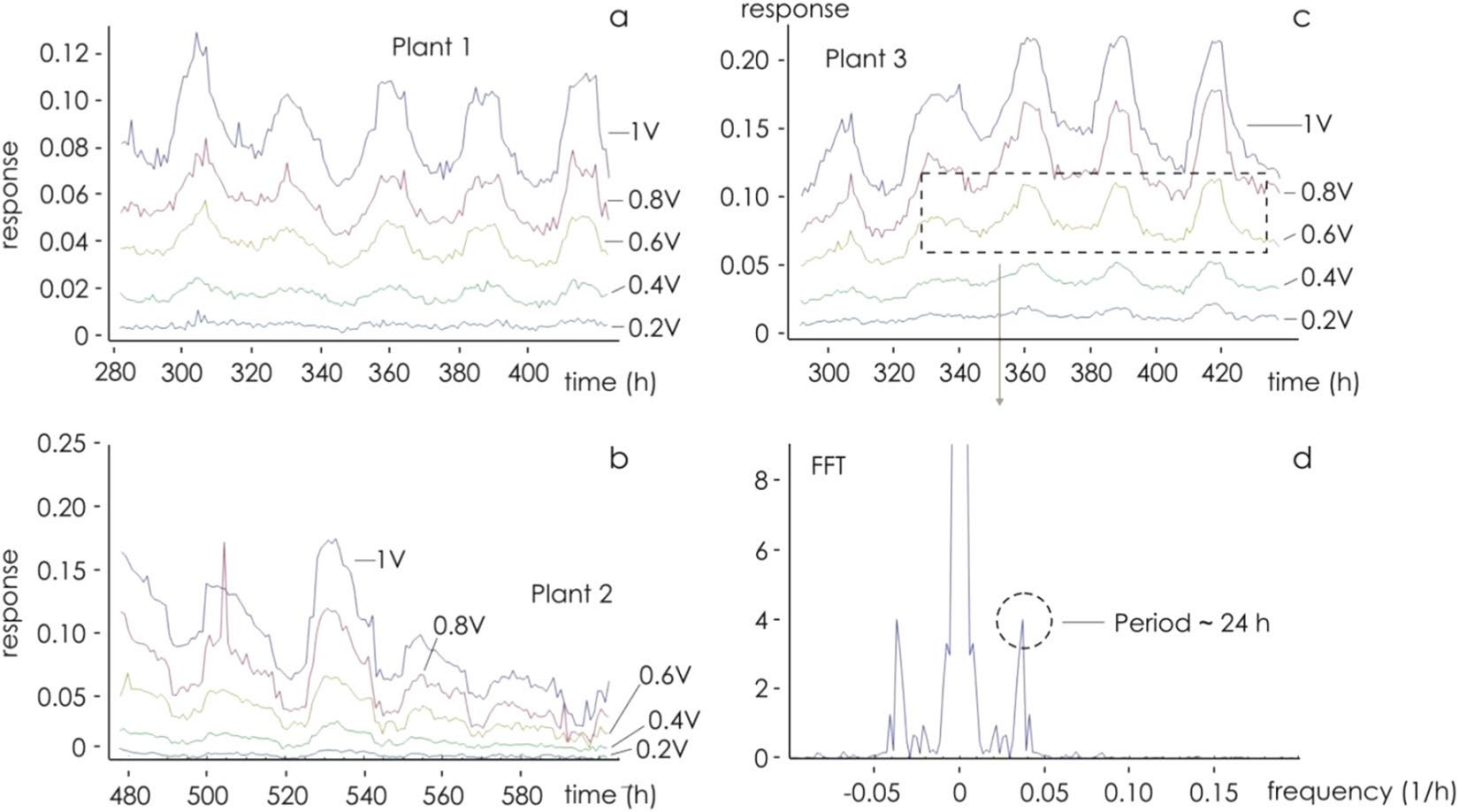
Variation of sensor response (R). (**a**–**c**) Bioristor response for plant 1, 2, and 3 at different V_gs_ bias, respectively; (**d**) Fast Fourier transform (FFT) of plant 3 response at V_gs_ = 1 highlighting the periodicity of the I_ds_ current.

The response of I_ds_ to variation in V_ds_ for a given V_g_ is illustrated in Fig. 2a; it behaves in the manner expected for a transistor placed within a conducting fluid. In particular, as the gate voltage (V_g_) was raised, I_ds_ decreased as a result of the de-doping of the conductive polymer, demonstrating that the plant sap was interacting with the device and that the sensor response was modulated by the ionic content. The time dependence of I_ds_ at a fixed V_ds_ as V_g_ was stepped from 0 to 1 V respectively is shown in Fig. 2b. The length of the cycle ensured that a value for the sensor response (R) could be derived every 24 min. The calculated response of R to an increase in V_g_ is illustrated in Fig. 2c; this confirmed that the device imposed the desired gating action and that ion diffusion through the plant sap was occurring. The behavior of R in response to variation in V_g_ over a 22 days period is shown in Fig. 3. The maximum response was obtained when V_g_ was set to 1 V. The variation in R for each of the three test plants is shown in Fig. 4a–c. The same periodicity was observed in each case: R increased during the dark period, and drop during the light period. The Fourier transformation of the responses clearly indicated that the driver of the periodicity was the circadian cycle (Fig. 4d). Time evolution of the response R is reported in Fig. 4 for three different plants (a–c) for different gate voltages. In the figure we reported examples extracted from larger data sets. At least 20 measurements per plant were repeated. For all reported cases, the response exhibits periodicity. Autocorrelation methods and fast Fourier transform analysis (Fig. 4d) indicate that the period is p_1_ = 23.95 ± 0.1 h, p_2_ = 24.0 ± 0.12 h, p_3_ = 24.12 ± 0.08 h, for the three plants. Pair-wise comparisons between means of different groups were performed using a Student’s t-test (two tailed, unpaired) and indicated that differences between means are not statistically significant for each couple of normally distributed populations. Thus main cycle is ~24 h for all considered cases and this suggests that the driver of periodicity is the circadian cycle.

These observations support bioristor effectiveness in measuring physiological changes within the plant sap. Indeed, the increasing of the sensor response in the night that drops during the day, is consistent with the transpiration oscillation^35^ and the circadian regulation of photosynthesis previously described^36,37^. Plant growth and development relies heavily on the movement of various ions through the plant sap^38^. The observed circadian variation in sap electrical conductivity was consistent with the literature describing the long distance transport of solutes through the xylem: concentrations are typically higher during the night than during the day^39^. Most of this difference is driven by rate of water movement within the plant, but an additional basis for the difference has been suggested as deriving from a greater rate of water extraction from the soil during the day, resulting in a higher degree of electrolyte dilution^32^.

### Parameterization of the outputs

**Figure 5:**
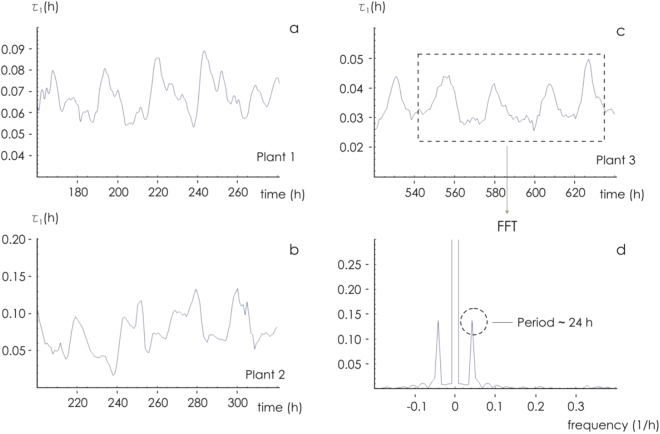
Characteristic time constants variations for V_gs_ = 1 V for: (**a**) Plant 1, (**b**) Plant 2 (**b**) and (**c**) Plant 3; (**d**) Fast Fourier transform (FFT) of time constants signal.

Upon the application of an external voltage across the device, I_ds_ varied over time from zero to a steady state value following a first order system dynamics (Fig. 2b). This behavior can be understood taking into account the equivalent circuit of the OECT that was already fully discussed^40^. This allowed a time constant (τ) to be calculated by fitting a non-linear curve to the data^41^. The value of τ is expected to depend on the nature of the electrolyte (atomic mass, net charge, diffusion coefficient), so reflects the ionic composition of the plant sap^41^. The τ values based on a V_g_ of 1 V for each of the three test plants monitored is shown in Fig. 5a–c. In each case τ varied periodically; the convolution with a periodic sinusoidal function with tunable period and fast Fourier transform analysis (FFT, Fig. 5d) yielded a periodicity value of 24 h, consistent with the value derived from the response sensor data shown in Fig. 3. Thus, both the intensity and dynamics of the current generated through the device followed a circadian rhythm.

**Figure 6:**
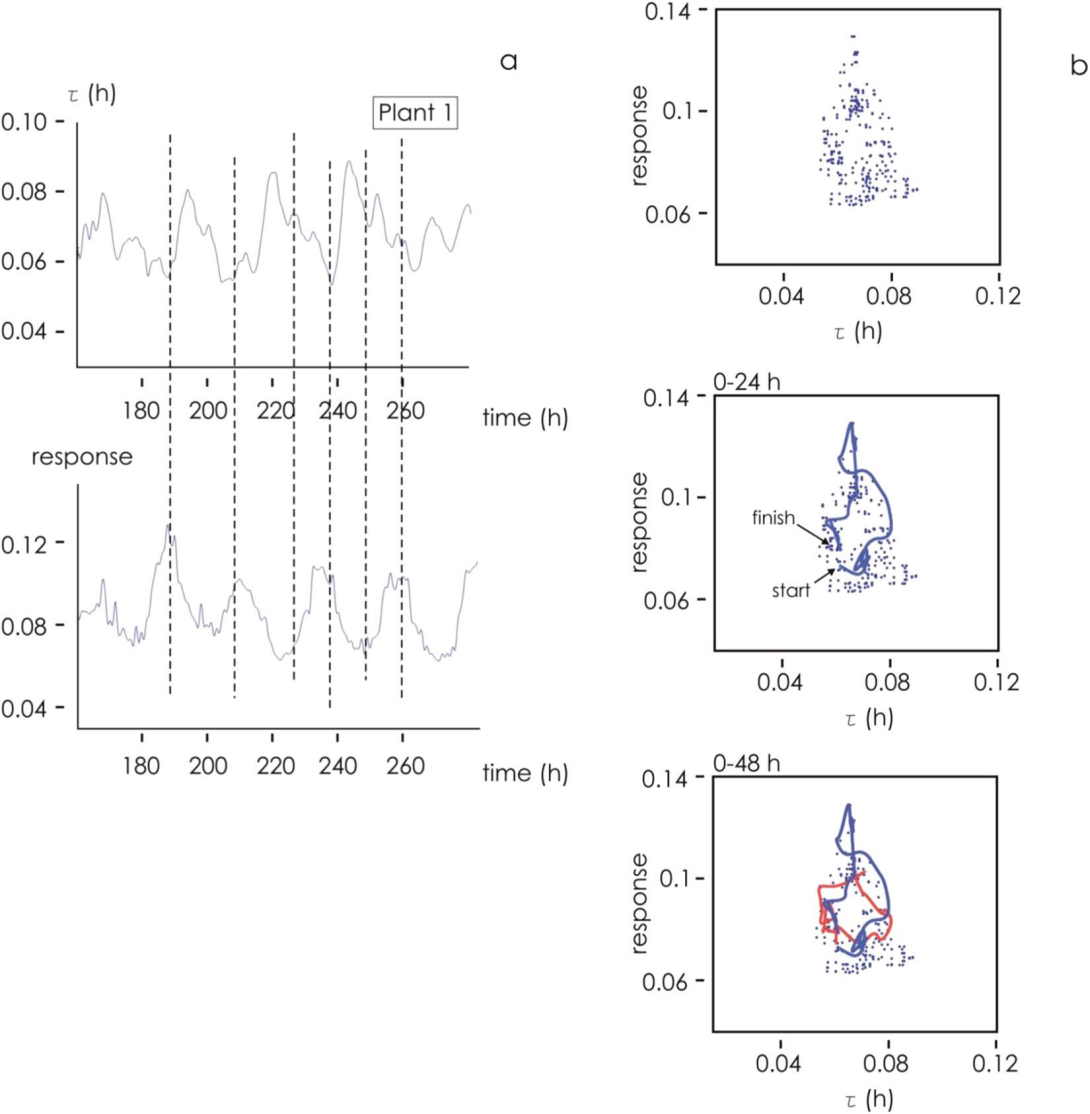
Parameterization of the output (**a**) time constants and sensor response as a function of time for plant 1 at V_gs_ = 1 V; (**b**) Phase diagram of sensor response as a function of time constants (top). Trajectories in the phase diagram for 0–24 h time intervals (middle) and 0–48 (bottom).

Figure 6a shows that τ and R are phase shifted: peak values of τ coincide with trough values of R and *vice versa*. The likely explanation for this asynchrony is that the greater the diffusivity of ions in solution, the higher is R (which is proportional to the number of ions collected by the gate electrode within a given time interval) and the smaller is τ (which is proportional to the time required by ions to reach their target)^40,41^. This suggests that R can be represented as a function of τ akin to the phase space diagrams used in dynamic systems. Assuming that the behavior of the OECT can be modeled by just two parameters (R and τ), each coordinate in the diagram describes the state of the plant at a specific time. Thus, the trajectory described by a sequence of coordinates can be used to describe the dynamic behavior of the system. The path followed in the R-τ plane is illustrated for Plant 1 in Fig. 6b. A whole revolution was completed in approximately 24 h, reflecting the circadian periodicity followed by the plant. The phase space diagram of the system is characterized by polar axial symmetry, which implies that R and τ are time locked: in the limit of signals with (i) a perfect harmonic wave-form, (ii) same frequency (period) and (iii) time lag (phase difference) of half-period, the appearance of the figure would be a perfect circumference. Any disruption in the symmetry of the path or deviations from a circle are indicative of an alteration in the plant’s physiological state. The form of the diagram suggests a lag between R and τ: if the curve takes the form of an ellipse with the major axis of the ellipse lying in the first and third quadrants of the R-τ plane, then changes in R precede those in τ, while the opposite is the case where the major axis lies in the second and fourth quadrants. The graphical representation of the OECT output can effectively signal variation in the physiological state of a plant, and can also reveal otherwise hidden patterns in the dynamics of the system as a whole.

### Biocompatibility of the bioristor

**Figure 7:**
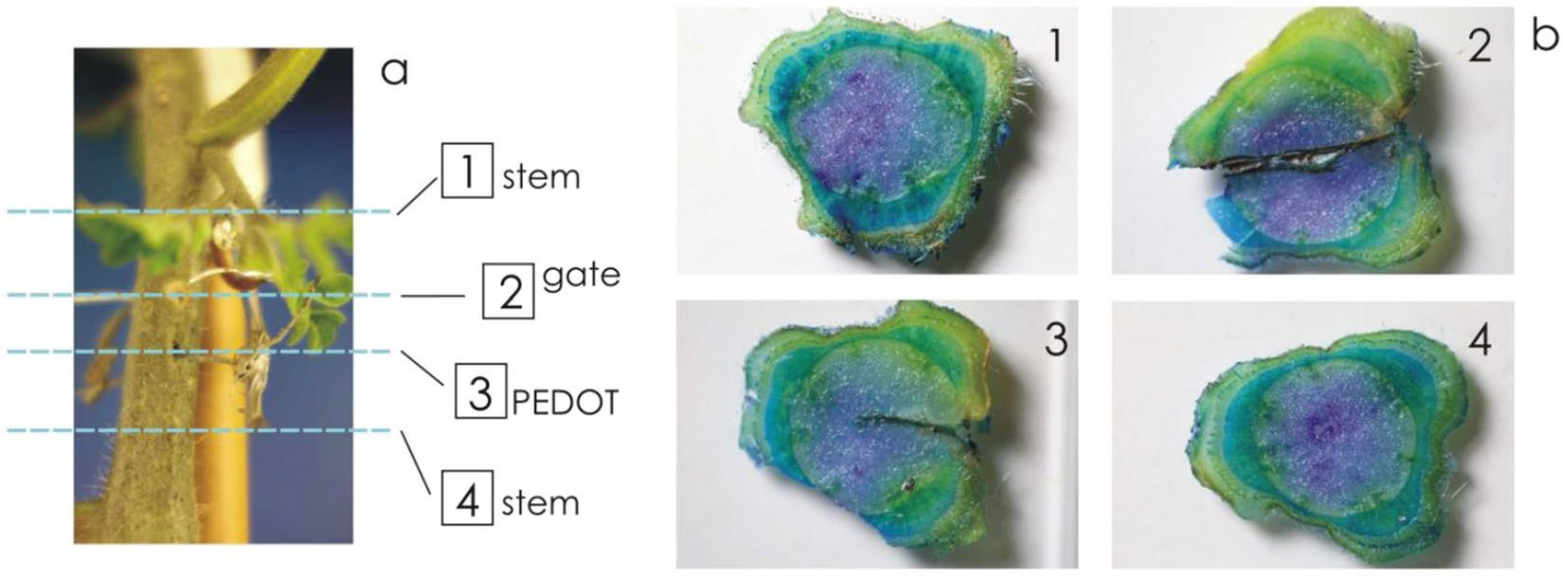
Biocompatibility of the bioristor. (**a**) Sketch of sections sampling along the plant stem; (**b**) Optical microscope image of plant section after 42 days of continuous sensor measurements: (b1) stem area on top, (b2) gate area, (b3) textile fiber area, (b4) steam area on bottom.

To verify that the sensor was fully biocompatible, stem sections of the three tomato plants were inspected 42 days after the sensors’ installation. Four different points along the stem were sampled (Fig. 7a): two were close to the gate electrode (Fig. 7b1,b2) and two close to the source/drain electrode (Fig. 7b3,b4). The analyses of the stem sections revealed that the vascular tissues path is not effected (data not shown) around the source/drain electrode and immediately before the gate, and that the presence of a damage necrotic layer of dead cells were observed mainly in correspondence of the gate electrode insertion point, and, with less extent, in correspondence of the pedot wire. The formation of necrotic cells surrounding the electrodes was not surprising since the insertion of the bioristor mimes a wounding event and the plant triggers a wide range of defense response to overcome the stress. However, plants have an incredible capacity to heal cuts and wounds by rapidly repairing tissues and vasculature even when two plants are cut and joint together^42^ and this is supported by the conventional use of grafting in horticulture and in fruit trees. The normal stem structure, morphology and vascular connections are completely restored immediately after the insertion points. Moreover, since the extent of the necrotic layer was reduced around the pedot wire, the silver wire would be better replaced with a textile-based electrode. There was no indication that the introduction of the bioristor altered the overall morphology of the stem and the presence of adventitious shoots was not detectable below the insertion points, indicating that the vascular connections were not interrupted. In addition, the plant growth was not affected, since the three plants equipped with the sensors were indistinguishable from other plants used as control (data not shown).

### Morphological and physiological analyses

To demonstrate that Bioristor does not alter the overall health status of the plant and does not affect morphology, water status, and photosynthetic machinery of 3 plants carrying the bioristor and 2 control plants without the sensor were compared as regard to morphological and physiological tests.

The plants were grown until full maturity stage, and no phenotypic differences were observed between plants carrying the bioristor and controls. All the plants regularly formed inflorescences and fruits; moreover, they showed an intermediate height of 60 cm and a number of leaves that varied between 8 and 9, true leaf in accordance with the tomato variety descriptors.

To test the leaves water status, the RWC was evaluated and no significant differences were observed between the wired plants (74% ± 3) and the controls (84% ± 7). The observed values fall within the range expected for a well irrigated plants indicating a high leaf hydration^43^.

When the chlorophyll content expressed in SPAD mean value (SV) was analyzed, again no significant differences between tomato plants carrying the sensor (42,2 ± 3,3) and controls (40 ± 6,8) were observed indicating a good status of the photosynthetic machinery^44^.

Taking all together these data in combination with the tissue biocompatibility analyses strongly supports the hypothesis that bioristor does not alter physiological processes in tomato plants.

## Discussion

Sensors constructed from textile material offer a number of advantages over those based on more conventional materials. Natural fibers are biomimetic structures, which are therefore more readily accepted by the plant. They can measure ion activity in the plant, yielding the best integration/interaction with the plant biology. Lastly, they enable a low cost means of monitoring the plant’s physiological state *in vivo* and in real time, which is not necessarily achievable using conventional materials or image-based remote and proximal sensors currently used in phenotyping platforms and for precision farming. The ability of the present textile-based OECTs to monitor the electrolyte content of the plant lies behind their naming as “bioristors”. The device is capable of transducing the plant’s physiological state and is readily adapted to continuously monitor it over a prolonged period. The development of a data analysis methodology which reduces the dynamic behavior of the currents into a phase space diagram based on R and τ was able to recognize the circadian nature of the plant’s sap electrolyte content. The device provides the means to monitor a plant’s physiological state in real time, and so can be potentially informative with respect to the early detection of drought stress or nutrient deficiency. This feature would be beneficial in the context of precision agriculture and as an integrative tool in plant phenotyping pipelines and represent a tool to address the needs of improvements in crop yield. Moreover, the so far developed sensors are not able to have a rapid response to plant changes and may lose information and data when plants experience rapid fluctuation of environment conditions triggering extremely rapid changing in growth and defence responses. The widespread use of this sort of technology could make a significant contribution to optimizing input usage and reducing water consumption, both of which are high priority goals in the context of achieving agricultural sustainability and maintaining producers’ income. Indeed, the possibility to monitor in continuous plant physiology opens new perspectives to dissect the mechanisms that take place in the plant during abiotic stress response, to understand where and when different ions are synthesized, allocated and translocated in normal and stress conditions, and, finally, to understand the links existing among genotype, environment, and phenotypes^45^.

## Methods

### Construction of the bioristor

The construction of the bioristors was adapted from^32^. Commercial cotton fibers were functionalized by soaking them for 5 min in aqueous poly(3,4-ethylenedioxythiophene) doped with polystyrene sulfonate (CleviosPH500, Starck GmbH, Munich, Germany), after which ethylene glycol (10% v/v) and dodecyl benzene sulfonic acid (12% v/v) were added. The fibers were then baked at 150 °C for 3 h. A treated fiber was inserted through the stem of a tomato plant, with a direct insertion and was cut to a length so that the ends protruded from opposite sides of the stem. The thread was connected on each end to a metal wire, forming the “source” and “drain” electrodes. The transistor device was completed by introducing a thin (diameter 500 µm) silver wire to act as a gate electrode (Fig. 1). The electrodes were connected to a NI USB-6343 multifunction I/O device (National Instruments, Austin, TX, USA) (Fig. S1).

### Signal acquisition

Tomato seeds were germinated in petri dishes at 25 °C, 50% relative humidity under a 16 h photoperiod, then transplanted into 1 L pots containing a 1:1 mixture of soil and peat. The plants were watered daily. When the plants had reached the sixth leaf stage (six weeks after germination), a device was inserted into each of three test plants (plants 1 to 3). A fourth device, which served as a reference, was connected to a 0.1 M NaCl solution held in a closed 2 ml microcentrifuge tube to avoid evaporation and therefore not subject to any changes in electrolyte concentration. Plants tracked for an extended time (42 days) were held at 24 °C and 50% relative humidity under a 16 h photoperiod. Measurements were performed, by applying a constant voltage (V_ds_) across the fiber and a positive voltage at the gate (V_g_); the behavior of the resulting current I_ds_ was monitored over time. The sensor response parameter R was expressed by R = (I_dS_ − I_dS0_)/I_dS0_, where I_ds0_ represented the current when V_g_ was zero. The response was proportional to the electrolyte concentration in the plant sap. This set-up allowed for the monitoring of the temporal variation in the plant sap’s electrolyte content, which is correlated to the plant’s physiological status.

The current transients were fitted to a single exponential curve to obtain the time constants at each setting of V_g_.

### Tissue staining

 Three plants with the integrated sensor and one control plant were analyzed at the end of the experiment for the biocompatibility following the protocol described in^46^. Four sections of stem tissue were prepared using a fresh razor blade. Toluidine Blue O (TBO, Sigma Aldrich, Milano, Italy) was used for the staining. It is a metachromatic stain that produces different colors depending on the polymer to which it adheres. Primary walls (parenchyma, collenchyma, and phloem) are purple and lignified secondary walls of xylem tracheids and vessels (a subtype of vascular tissue) and sclerenchyma are blue, while some other cells may take on a greenish color^46^. Pictures were acquired with a digital camera equipped with a macro lens.

### Morphological and physiological analyses

The 3 tomato plants carrying the bioristor where grown contemporary with 2 control plants that do not carry the sensor; all plants were evaluated for the morphology traits were visually scored according to the tomato descriptors.

The Relative Water Content (RWC) was evaluated as follows. Fresh weight (FW) was immediately recorded (time 0), and then seedlings were placed on dry paper for 24 h at room temperature and the weight (W24) was measured again. After, leaves were soaked in distilled water for 24 h at room temperature in darkness and the turgid weight (TW) was recorded. Finally, total dry weight (DW) was recorded after drying for 24 h at 80 °C. The RWC was calculated according to Barrs *et al*. (1962)^47^. Chlorophyll content measurements were performed by using the SPAD502 meter. Measurements from each plant, 10 leaves of varying age and color were selected for measurements made under diffuse lighting^44^. The relative SPAD-502 meter value is proportional to the chlorophyll content and reported. Every leaf measurement was an average of 10 SPAD-502 readings.

All data were analyzed statistically applying the student’s t-test and standard error was calculated between replicas.

### Data availability

The data supporting the findings of this study are available from the corresponding authors upon request.

## Electronic supplementary material

Supplementary Figure 1
