# Supplementary material for: An in vivo biosensing, biomimetic electrochemical transistor with applications in plant science and precision farming
Source: Sci Rep. 2017 Nov 23;7:16195. doi: 10.1038/s41598-017-16217-4 (PMC5700984; doi:10.1038/s41598-017-16217-4)
Supplement: Supplementary file 1 — Supplementary Figure 1 [file 41598_2017_16217_MOESM1_ESM.pdf]

# An *in vivo* biosensing, biomimetic electrochemical transistor with applications in plant science and precision farming

Nicola Coppedè[1], Michela Janni[1,4], Manuele Bettelli[1], Calogero Leandro Maida[2], Francesco Gentile[3], Marco Villani[1], Roberta Ruotolo[2], Salvatore Iannotta[1], Nelson Marmiroli[2], Marta Marmiroli[2], Andrea Zappettini[1]

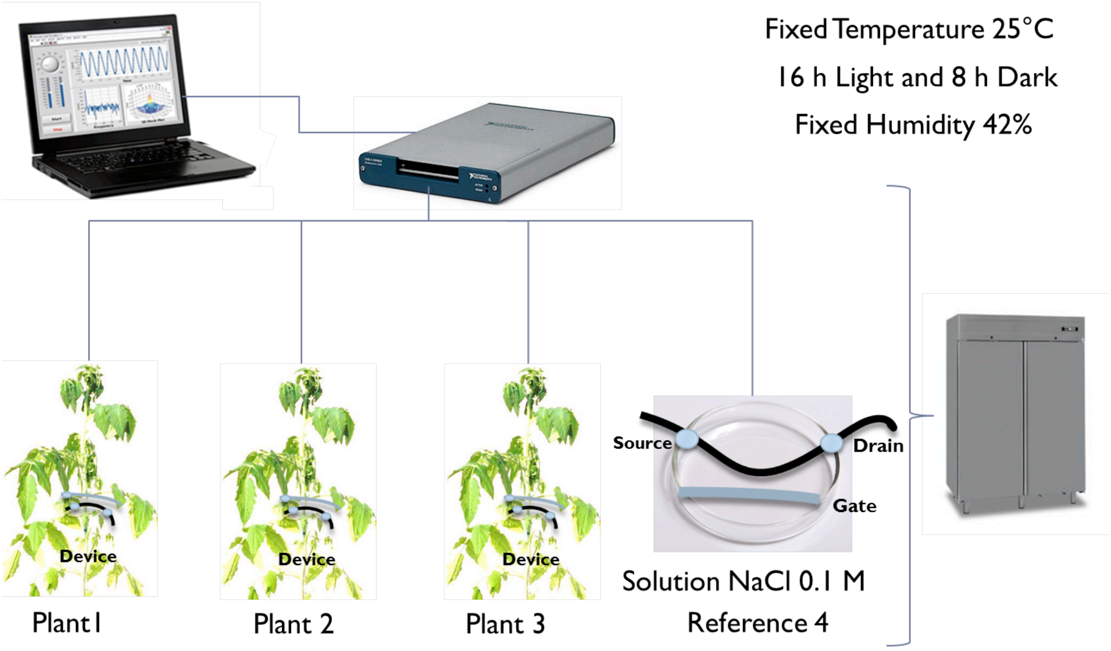


Supplementary Figure 1: Scheme of the measuring apparatus: on top the reading board controlled by the computer and connected to the bioristors mounted on three plants, and a reference device in saline solution. Plants and reference device are stored in a controlled growth chamber (on the right).
